# Genome-Wide Identification and Low Temperature Responsive Pattern of Actin Depolymerizing Factor (ADF) Gene Family in Wheat (*Triticum aestivum* L.)

**DOI:** 10.3389/fpls.2021.618984

**Published:** 2021-02-24

**Authors:** Ke Xu, Yong Zhao, Sihang Zhao, Haodong Liu, Weiwei Wang, Shuhua Zhang, Xueju Yang

**Affiliations:** ^1^State Key Laboratory of North China Crop Improvement and Regulation, Hebei Agricultural University, Baoding, China; ^2^Cangzhou Academy of Agriculture and Forestry Sciences, Cangzhou, China

**Keywords:** actin depolymerizing factor, wheat (*Triticum aestivum* L.), low temperature, genome-wide identification, transgenic Arabidopsis

## Abstract

The actin depolymerizing factor (ADF) gene family, which is conserved in eukaryotes, is important for plant development, growth, and stress responses. Cold stress restricts wheat growth, development, and distribution. However, genome-wide identification and functional analysis of the ADF family in wheat is limited. Further, because of the promising role of ADF genes in cold response, there is need for an understanding of the function of this family on wheat under cold stress. In this study, 25 ADF genes (*TaADFs*) were identified in the wheat genome and they are distributed on 15 chromosomes. The TaADF gene structures, duplication events, encoded conversed motifs, and *cis*-acting elements were investigated. Expression profiles derived from RNA-seq data and real-time quantitative PCR analysis revealed the tissue- and temporal-specific TaADF expression patterns. In addition, the expression levels of *TaADF13/16/17/18/20/21/22* were significantly affected by cold acclimation or freezing conditions. Overexpression of *TaADF16* increased the freezing tolerance of transgenic *Arabidopsis*, possibly because of enhanced ROS scavenging and changes to the osmotic regulation in cells. The expression levels of seven cold-responsive genes were up-regulated in the transgenic Arabidopsis plants, regardless of whether the plants were exposed to low temperature. These findings provide fundamental information about the wheat ADF genes and may help to elucidate the regulatory effects of the encoded proteins on plant development and responses to low-temperature stress.

## Introduction

Actin depolymerizing factor (ADF), which is a conserved protein family with a low molecular weight (15–22 kDa) (Staiger et al., 1997) in eukaryotic cells, is crucial for regulating the reorganization and rearrangement of the actin cytoskeleton (Maciver and Hussey, 2002). This protein was first identified in chick embryo brains (Bamburg et al., 1980), but it has since been detected in various plants (Gungabissoon et al., 1998; Smertenko et al., 1998; Dong et al., 2001; Wang et al., 2009; Fu et al., 2014). As a member of the actin-binding protein family, ADF remodels the actin cytoskeleton via the transition between F-actin and G-actin states (Du et al., 2016). The actin cytoskeleton influences cellular architecture as well as diverse processes, including the expression of cell polarity, cell expansion and division, intracellular transport, pathogen perception, abiotic stress responses, and signal transduction (Fowler and Quatrano, 1997; Drobak et al., 2004; Porter and Day, 2016; Allard et al., 2020).

Because of the various actin cytoskeleton functions affecting plant development and responses to stimuli, ADF proteins in higher plants are important for various cellular activities. For example, ADFs are involved in pollen development and pollen tube growth in *Arabidopsis* (Daher and Geitmann, 2012; Zheng et al., 2013), maize (Lopez et al., 1996), and tobacco (Chen et al., 2002). Previous studies revealed that a lack of *AtADF9* results in early flowering (Burgos-Rivera et al., 2008) and knockout of *AtADF4* increases the length of hypocotyls and epidermal cells (Henty et al., 2011). In cotton, *GhADF1* is involved in fiber elongation and secondary cell wall formation (Wang et al., 2009). Additionally, the expression patterns of ADF genes appear to be tissue-specific. Earlier research indicated that *OsADF2/4/5* are expressed in roots, stems, blades, sheaths, spikelets and seeds persistently, whereas *OsADF9* expression is specific to spikelets at the heading stage in rice (Huang et al., 2012). The *GhADF6/8* genes are preferentially expressed in petals, while *GhADF7* is highly expressed in anthers of cotton (Li et al., 2010). Moreover, ADF proteins reportedly have a vital role in responses to abiotic and biotic stresses in *Arabidopsis*, wheat, rice, and other species (Ouellet et al., 2001; Huang et al., 2012; Fu et al., 2014; Sengupta et al., 2019). The overexpression of *OsADF3* enhances the drought tolerance of transgenic *Arabidopsis* seedlings (Huang et al., 2012). The expression of wheat *TaADF4* is induced by heat, but down-regulated by low temperatures or salt stress (Zhang et al., 2017). In contrast, *TaADF3* expression is significantly up-regulated under cold conditions and water deficiency treatment, but is relatively un-affected by wounding or salt stress (Tang et al., 2016). Both *TaADF4* and *TaADF7* enhance the resistance of wheat plants to a *Puccinia striiformis* f. sp. *tritici* (*Pst*) infection, whereas *TaADF3* has the opposite effect (Fu et al., 2014; Tang et al., 2016; Zhang et al., 2017).

Low-temperature stress is a key factor influencing the growth, yield, and distribution of wheat plants (Song et al., 2017). An exposure to low but nonfreezing temperatures (i.e., cold acclimation) is crucial for the freezing tolerance of over-wintering plants (Thomashow, 2001; Sung and Amasino, 2005). Although there are relatively few reports describing ADFs in wheat exposed to abiotic stress, two earlier investigations proved that ADF production is induced at low temperatures (Danyluk et al., 1996; Ouellet et al., 2001). During the cold acclimation process, the depolymerization of microtubules and actin filaments increases the fluidity of the plasma membrane (Danyluk et al., 1996). However, the molecular mechanism regulating the depolymerization of microtubules and actin filaments under cold conditions remains unknown. Thus, clarifying the relationship between ADF proteins and freezing tolerance response is warranted.

The development and application of genome sequencing technology has led to the identification of ADF genes in the genomes of several plant species, including rice, maize, tomato, and *Arabidopsis* (Feng et al., 2006; Khatun et al., 2016; Huang et al., 2020). The availability of a sequenced ‘Chinese Spring’ genome has helped to facilitate the genome-wide analysis of gene families in wheat (Hu et al., 2018; International Wheat Genome Sequencing Consortium (IWGSC), 2014; Liu et al., 2019; Zhou et al., 2019). However, to the best of our knowledge, there are no reports regarding the genome-wide identification and characterization of wheat ADF genes or the wheat ADF gene expression profiles in various tissues and in response to low-temperature stress. In this study, we identified 25 wheat ADF genes using the ‘Chinese Spring’ genome sequences (IWGSC, RefSeq V1.1), after which we analyzed the ADF gene structures and the encoded conserved motifs, determined the genomic locations and duplication events of the ADF genes, and predicted the putative *cis*-acting elements. Additionally, ADFs in *Triticum dicoccoides*, *Hordeum vulgare*, *Triticum turgidum*, *Triticum urartu*, and *Aegilops tauschii*, were identified and used along with the wheat ADFs to construct a phylogenetic tree. To further investigate the function of ADF in wheat, we analyzed the expression profiles of these genes in different tissues, as well as in response to cold stress. Furthermore, we examined the effects of *TaADF16* overexpression on the freezing tolerance of *Arabidopsis*. The results of this study will enhance our understanding of the wheat ADF gene family and provide the basis for future investigations of ADFs in wheat.

## Materials and Methods

### Identification of ADF Genes in Wheat

The genome and protein sequences data of wheat were downloaded from Ensembl Plants database^1^. The actin-depolymerizing factor homology domain (ADF-H domain, with Pfam: PF00241) obtained from PFAM database^2^ was employed as a query for Hidden Markov Model (HMM) search using HMMER3.0 with a pre-defined threshold of *E* value ≤ 1e^–10^. The results obtained were used to construct a wheat-specific ADF HMM profile by hmm-build program, and the second HMM search was used to remove the redundant sequences among the identified ADF proteins with an *E* value ≤ 1e^–10^. After manual corrections applied as needed, the NCBI-CDD web server^3^, SMART database^4^ and Pfam database (see foot note 2) were used to further confirm the ADF_H domain in the putative ADF protein sequences. The biochemical parameters of TaADF proteins, such as isoelectric points (pI), molecular weights (MW), instability index (II), aliphatic index (AI) and calculated grand average of hydropathy index (GRAVY) of the putative ADF proteins were calculated using the ExPASy online protParam tool^5^ (Artimo et al., 2012). The prediction of subcellular location of the identified TaADFs were performed using Plant-mPLoc^6^ (Chou and Shen, 2010). Alignment analysis of TaADF proteins was performed using MEGA 7.0, and visualized by Jalview v2.11.1.3 (Waterhouse et al., 2009).

### Phylogenetic Tree Construction of Wheat and Other Eight Species

The phylogenetic tree was performed using the neighbor joining (NJ) method in MEGA 7.0, with 1,000 bootstrap replicates. Sequences of ADF proteins from select species were identified based on the corresponding genome (Supplementary Table 1) as described above. The accuracy of identified ADF was confirmed with Ensembl Plants^1^ and Uniport database^7^.

### Gene Structure, Motif Analysis and *Cis*-acting Elements of TaADF Gene Family

The exon/intron structures of TaADFs were constructed by gene structure display server (GSDS) program^8^ using the CDS and corresponding genomic sequences retrieved from the Ensemble plants database. Conserved motifs of TaADF discover were predicted using the Multiple Expectation Maximization for Motif Elication (MEME) 4.12.0^9^, with the following parameters: maximum number of 20 motifs and optimum motif widths of 6-100 residues. The 1500 bp upstream of the transcription start site (−1) of all identified TaADF transcripts was extracted as promoter to predict *cis*-acting elements using Plant CARE^10^.

### Chromosomal Localization, Gene Duplication and Calculating Ka/ks Values of TaADF

All the *TaADF* genes were mapped to wheat chromosomes based on physical location information from the database of wheat genome. The gene duplication in the wheat genome were analyzed with Multiple Collinearity Scan toolkit (MCScanX) (Wang et al., 2012) and visualized with Circos tool (Krzywinski et al., 2009). The calculation of ka and ks substitution of each duplicated *TaADF* genes were performed using KaKs_Calculator 2.0 (Wang et al., 2010). The syntenic maps between wheat and other species were constructed using the python version of MCScanX^11^.

### Expression Pattern of *TaADF* Genes in Different Tissues and Development Stages

The expression patterns of identified *TaADF* genes in different tissues and development stages were analyzed based on the publicly available wheat RNA-Seq datasets obtained from wheat eFP Browser^12^ (Ramírez-González et al., 2018). The expression levels were summarized as transcripts per million (TPM), and a heatmap of *TaADF* tissues-specific expression were conducted with R packages (Pheatmap and Stats).

### Plant Materials and Growth Conditions of Wheat

For expression analysis of *TaADFs* of different tissues, seeds of wheat ‘Chinese Spring’ were grown under 20°C with a 12/12 h photoperiod/dark in glass dish for 15 day (three-leaf stage). Roots and leaves were collected from five seedlings. For low temperature treatment, the wheat seedlings of ‘Jing 411’ were cultivated in the incubator at 20°C with a 12/12 h photoperiod/dark period for 15 days until three-leaf stage (TL), which was followed with different temperature treatments: 4°C for cold acclimation (CA), 20°C for un-cold acclimation (UCA). After 28 days, the CA and UCA seedlings were exposed to −5°C for 1 day (cold acclimation and freezing, CAF; un-cold acclimation and freezing, UCAF). All the samples were immediately frozen in liquid nitrogen and stored at −80°C. Three biological replications were performed.

### RNA Extraction, RNA-Seq and RT-PCR Validation

The samples were subjected to total RNA extraction using a Trizol Reagent (Invitrogen, Carlsbad, CA, United States). The analysis of RNA-seq for low temperature treatment was based on our previous study (Zhao et al., 2019b), we re-analyzed the data based on reference genome of ‘Chinese Spring’ genome (IWGSC: RefSeq V1.1). Sampled crowns from TL, CA, UCA, CAF, and UCAF were used for RNA-seq analysis. Using the DESeq R package, the differently expressed genes (DEGs, | log2 (fold change)| > 1 and *p* < 0.05) were analyzed.

cDNA products were subjected to RT-PCR analysis, in which, *TaGAPDH* and *TaTEF1*-α were used as double internal reference genes for wheat. RT-PCR was performed with BCS Wiz SYBR Green RT-PCR Master Mix and the QuantStudio 5 Real-time PCR system (Applied Biosystems, Malham, MA, United States). The following amplification protocol was used: first step, 95°C for 30 s; second step, 40 cycles of 95°C for 5 s and 60°C for 30 s; final step, 95°C for 15 s, 60°C for 1 min, 95°C for 15 s, and 50°C for 30 s. The relative expression were calculated with 2^–ΔΔCt^ method. Three biological replications were performed (each biological replication for three technical). Specific primers used in this study are shown in Supplementary Table 2.

### Subcellular Localization of TaADFs

The coding sequence (CDS) of *TaADF11*, *TaADF14*, *TaADF15*, and *TaADF16* was cloned into the pBWA(V)HS-ccdb-GLosgfp vector containing the cauliflower mosaic virus 35S (CaMV 35S) promoter, respectively. Subsequently, the control plasmid and fusion plasmids were transiently expressed in *A. thaliana* protoplasts. Then, the transformed protoplasts were incubated for 24 h at 22°C darkness. Finally, green fluorescent protein (GFP) fluorescence signals were observed using Nikon C2-ER confocal laser scanning confocal microscope. The specific primers containing the restriction site are shown in Supplementary Table 2.

### Overexpression of *TaADF16* Genes in Arabidopsis and Freezing Tolerance Assay

The CDS of *TaADF16* (Gene id: TraesCS5A02G478500), was amplified by PCR with gene-specific primer (F: 5′-GG AGAGGACACGCTCGAGATGACTTTATCTCGCCGACATG-3′ and R 5′-TTAAAGCAGGACTCTAGATTAGGTGGTGTAGT CCTTGAGGAT-3′) and then cloned into the pART-CAM vector controlled by the CaMV *35S* promoter. The *35S:TaADF16* plasmid were transformed into *Agrobacterium tumefaciens* GV3101 and then transformed into *Arabidopsis*. Seeds of T0 transgenic plants were selected on MS medium containing 100 μg/ml kanamycin and further confirmed by PCR. T3 homozygous of *Arabidopsis* transgenic plants was used for freezing tolerance assay.

For freezing tolerance analysis in WT and OE lines, all the seeds were planted in plastic pots filled nutrient soil for three weeks, with 14/10 h photoperiod and temperature at 22°C. Three weeks old seedlings were transfered to −5°C for 4 h for freezing stress and ion leakage was determined. For recovery treatment, the plants after freezing were placed in the dark at 4°C for 12 h followed by 4 day at 22°C and the survival rates was determined. Photos were taken to record the growth phenotype before treatment and after recovery. Leaves of plants after 4°C for 24 h were collected for analysis of POD and SOD activities, MDA and soluble sugar content as described by Li et al. (2000). Seven cold responsive genes were selected for RT-PCR assay. *AtTUB2* and *AtUBQ10* of *Arabidopsis* was used as double internal reference gene. Three biological replications were carried out for each sample. The primers of the genes for RT-PCR are listed in Supplementary Table 2.

### Statistical Analysis

Data were statistically processed by the SPSS 25.0 and Graphpad Prism 8 software. The mean value ± standard deviation (SD) of at least three replicates for each sample are presented. Statistical significance was assessed by Student’s *t*-test between control and treatment.

## Results

### Identification of ADF Gene Family Members in Wheat

A total of 25, 18, 8, 5, 12, and 11 non-redundant putative ADF genes were identified in wheat (*TaADF1*–*TaADF25*), *T*. *dicoccoides* (*TdADF1*–*TdADF18*), *Ae*. *tauschii* (*AetADF1*–*AetADF8*), *T. urartu* (*TuADF1–TuADF5*), *T. turgidum* (*TtADF1*–*TtADF 12*), and barley (*HvADF1*–*HvADF11*), respectively (Table 1 and Supplementary Table 3). Of the encoded TaADF proteins, TaADF4/8 and TaADF22 were, respectively, revealed as the shortest (132 amino acids) and longest (235 amino acids). The molecular weights (MW) of the TaADF proteins ranged from 15.30 to 25.91 kDa and the isoelectric points (pI) ranged from 4.39 to 8.74. The GRAVY values (<0) reflected the hydrophilicity of the TaADF proteins. An analysis of the instability index suggested that 16 proteins (64%) may be unstable (instability index > 40) and nine proteins (36%) are probably stable (instability index ranging from 30.91 to 38.53). The aliphatic index, which ranged from 62.52 to 79.72, indicated the thermal stability of TaADF proteins.

**TABLE 1:** Description of actin depolymerizing factor (ADF) genes identified from the wheat database.

**Gene name**	**Gene ID**	**Physical Position**			**Information of Proteins**						
		**Chr**	**Start Position**	**End Position**	**ProteinLength**	**MW (Da)**	**pI**	**Instability Index**	**Aliphatic Index**	**GRAVY**	**Subcelluar Location**
*TaADF1*	TraesCS1A02G183100	1A	331517066	331518825	150	17310.45	5.08	42.61	72.73	−0.48	Cytoplasm
*TaADF2*	TraesCS1B02G197600	1B	355155044	355157167	150	17368.48	4.97	42.11	72.73	−0.50	Cytoplasm
*TaADF3*	TraesCS1D02G186600	1D	257474295	257475731	151	17493.70	4.97	40.05	76.75	−0.44	Cytoplasm
*TaADF4*	TraesCS2A02G410700	2A	668242515	668243490	132	15318.39	5.57	42.26	67.95	−0.47	Cytoplasm
*TaADF5*	TraesCS2A02G227300	2A	239381406	239386945	139	16142.22	5.75	38.25	62.52	−0.58	Cytoplasm
*TaADF6*	TraesCS2B02G255200	2B	290519507	290523695	139	16112.19	5.75	37.03	62.52	−0.58	Cytoplasm
*TaADF7*	TraesCS2B02G429500	2B	616934383	616935480	139	15918.01	5.87	37.09	65.25	−0.45	Cytoplasm
*TaADF8*	TraesCS2D02G408100	2D	523028514	523029011	132	15304.36	5.57	47.62	67.95	−0.47	Cytoplasm
*TaADF9*	TraesCS2D02G235200	2D	220670605	220675415	139	16168.30	5.75	42.80	65.32	−0.55	Cytoplasm
*TaADF10*	TraesCS4A02G071400	4A	68688963	68691716	143	16529.07	8.74	53.59	73.64	−0.35	Cytoplasm
*TaADF11*	TraesCS4B02G227300	4B	475810763	475813477	143	16529.07	8.74	53.59	73.64	−0.345	Cytoplasm
*TaADF12*	TraesCS4D02G228100	4D	387441390	387445034	143	16543.10	8.74	53.69	73.64	−0.35	Cytoplasm
*TaADF13*	TraesCS5A02G478800	5A	651858719	651859682	147	15800.62	4.48	35.58	79.05	−0.15	Cytoplasm
*TaADF14*	TraesCS5A02G416100	5A	605100341	605103635	145	16748.06	5.45	43.99	71.31	−0.42	Cytoplasm
*TaADF15*	TraesCS5A02G478900	5A	651982952	651984951	138	16084.13	5.65	38.53	63.62	−0.66	Cytoplasm
*TaADF16*	TraesCS5A02G478500	5A	651826274	651827210	200	22273.65	5.03	53.11	67.30	−0.56	Cytoplasm
*TaADF17*	TraesCS5B02G491800	5B	659983832	659984917	147	15824.67	4.60	34.84	79.05	−0.19	Cytoplasm
*TaADF18*	TraesCS5B02G491500	5B	659769899	659770854	142	15751.54	4.39	46.02	79.72	−0.32	Cytoplasm
*TaADF19*	TraesCS5D02G492400	5D	525658928	525661018	138	16084.13	5.65	38.53	63.62	−0.66	Cytoplasm
*TaADF20*	TraesCS5D02G423800	5D	483645409	483648797	145	16802.05	5.26	44.77	71.31	−0.46	Cytoplasm
*TaADF21*	TraesCS5D02G492300	5D	525602262	525603227	147	15886.80	4.78	30.91	75.71	−0.21	Cytoplasm
*TaADF22*	TraesCS5D02G491900	5D	525471415	525472578	235	25906.92	5.43	58.10	68.51	−0.50	Cytoplasm. Nucleus.
*TaADF23*	TraesCS6A02G247000	6A	457685808	457687435	139	15979.21	6.33	41.02	68.71	−0.43	Cytoplasm
*TaADF24*	TraesCS6B02G277600	6B	502483095	502484438	145	16584.01	6.33	37.25	68.55	−0.36	Cytoplasm
*TaADF25*	TraesCS6D02G229200	6D	320228058	320229328	139	15997.29	5.59	47.70	69.42	−0.38	Cytoplasm

*Chr, Chromosome; MW, Molecular weight (Da); pI, Isoelectric point; GRAVY, Grand average of hydropathicity.*

### Analysis of the Chromosomal Locations and Duplication of *TaADF* Genes

**FIGURE 1:**
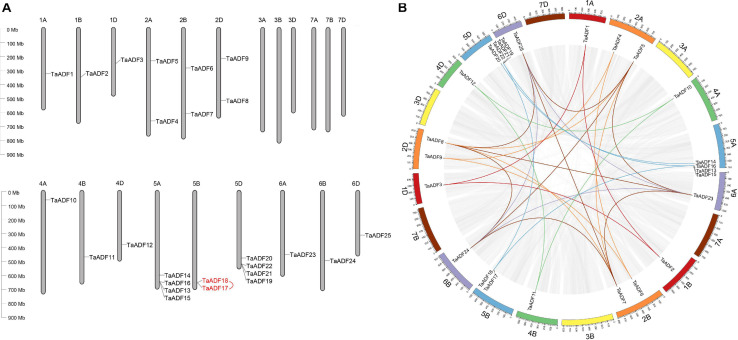
Chromosomal location and duplication events of ADF in wheat. **(A)** Chromosomal location of ADF genes in wheat. Red line between genes indicate tandem duplication of *TaADF* genes. **(B)** Segmental duplication in wheat genome. Gray lines indicate all synteny blocks in the wheat genome, and the different colors represent seven homeologous group of wheat chromosomes. Homologous genes of each group are linked by lines with corresponding color.

The results of the chromosomal localization and collinear analysis revealed that the *TaADF* genes are unevenly distributed on 15 chromosomes, with the number of *TaADF* genes on each chromosome ranging from 1 (1A, 1B, 1D, 4A, 4B, 4D, 6A, 6B, and 6D) to 4 (5A and 5D) (Figure 1A). Nine, seven, and nine *TaADF* genes were detected in the A, B, and D sub-genomes, respectively, implying some of the *TaADF* genes in the B sub-genome may be lost during evolution. These lost ADF genes in 5B may have a redundant function with the ADF genes on 5A or 5D, with a low purifying selection, they were finally lost during evolution. We found 7 homologous gene groups with a copy on each of A, B and D sub-genomes, and 2 gene pairs had two homologous genes on A and D sub-genomes. In addition, the homologous *TaADF* genes shared high protein sequence similarity, with a range of 92.5% (*TaADF16-5A*, *TaADF18-5B*, *TaADF22-5D*) to 100% (*TaADF15-5A* and *TaADF19-5D*) (Supplementary Table 4). Gene duplication events affected the *TaADF* genes on 15 chromosomes. Thirty-one segmental duplications (Figure 1B) and one tandem duplication (Figure 1A) were detected. The rates of non-synonymous (Ka) and synonymous (Ks) nucleotide substitutions were calculated to evaluate the selection pressure on the *TaADF* gene duplication events (Supplementary Table 5). The Ka/Ks ratios for the 32 duplicated pairs were less than 1.00, implying the wheat ADF genes evolved under strong purifying selection.

### Systematic Evolutionary Relationships Among ADF Members in Wheat and Eight Other Plant Species

**FIGURE 2:**
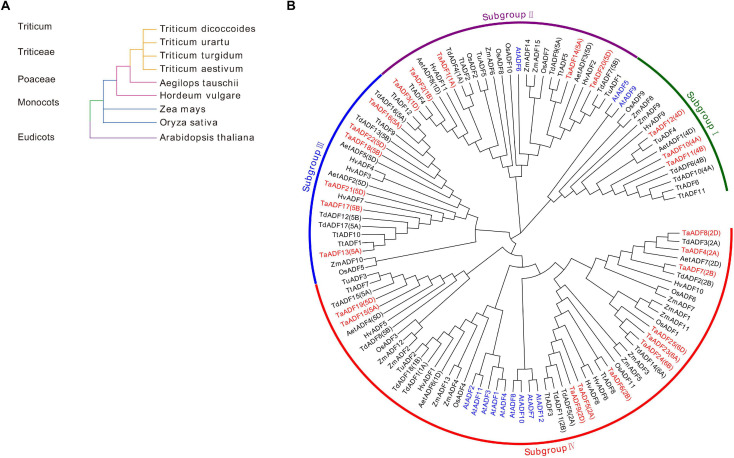
Systematic evolutionary relationships of ADF in wheat and eight other species. **(A)** Evolution relationship among the nine species; **(B)** Phylogenetic tree of ADF family members in wheat and eight other species. ADF proteins in *Triticum aestivum*. L., *Oryza sativa*, *Arabidopsis thaliana*, *Zea mays*, *Hordeum vulgare*, *Triticum turgidum*, *Triticum urartu*, *Triticum dicoccoides* and *Aegilops tauschii* are prefixed by Ta, Os, At, Zm, Hv, Tt, Tu, Td, and Aet, respectively. ADF proteins in wheat and *Arabidopsis* are marked in red and blue, respectively. The chromosome locations of ADF in *Triticum aestivum*. L, *Triticum turgidum*, and *Aegilops tauschii* were provided in the bracket followed with each gene name.

To investigate the evolutionary relationships and characteristics of the ADF genes, 117 ADF proteins from wheat and other species were used to construct a phylogenetic tree (Figure 2 and Supplementary Table 3). The evolutionary relationships between wheat and eight other species were determined (Figure 2A). The phylogenetic tree revealed that the ADF genes can be classified into four main groups, with each clade consisting of 15–56 members (Figure 2B and Supplementary Table 6). More specifically, 3, 5, 6, and 11 TaADF are clustered in Groups I, II, III, and IV, respectively.

**FIGURE 3:**
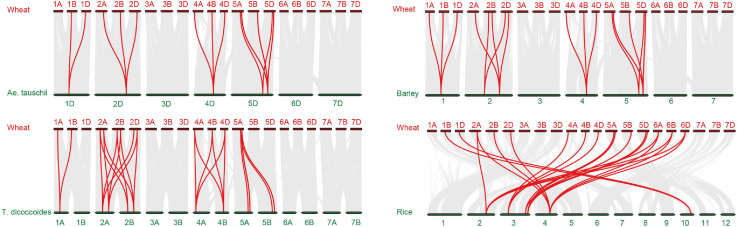
Synteny analysis of *ADF* genes between wheat and other four species. Gray lines in the background and red lines between different species indicate the collinear blocks and syntenic *ADF* pairs between wheat and other species, respectively.

To more thoroughly determine the phylogenetic mechanisms of *TaADF* genes, we examined the synteny between wheat and other four gramineous species, including *Ae. tauschii*, *T. dicoccoides*, barley, and rice. A total of 16, 26, 18, and 22 orthologous gene pairs between hexaploid wheat (*T. aestivum*) and other species (*Ae. tauschii*, *T. dicoccoides*, barley, and rice) were identified (Figure 3 and Supplementary Table 7). Some collinear pairs (with eight *TaADF* genes) were identified in all of the four syntenic maps, suggesting that these orthologous pairs were relatively well conserved during the evolution of gramineous species. Each of ADF genes in 2A, 2B, 4A, 4B of tetraploid wheat showed synteny to several ones in hexaploid wheat. However, some orthologous gene pairs were only identified between chromosome 5A (or 5B) of *T*. *dicoccoides* and 5A of hexaploid wheat, but not 5B of hexaploid wheat, which may be due either to the quality of the genome assembly or the gene deletion or chromosomal recombination during evolution and polyploidization.

### Structural Characterization of TaADF Genes in Wheat

**FIGURE 4:**
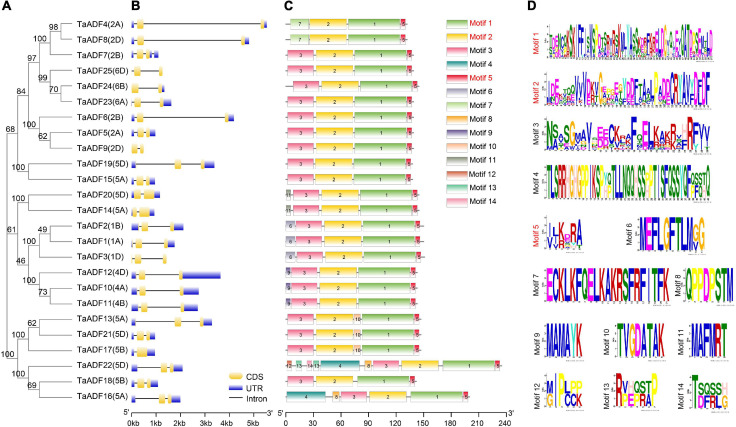
Phylogenetic relationship, exon-intron structures and motif composition of TaADFs. **(A)** Phylogenetic relationship of TaADFs. **(B)** Exon-intron structures of *TaADF* genes. Exons-intron are indicated by wide color bar and black line, respectively; **(C)** Distribution of conserved motifs of TaADFs predicted by MEME tool. **(D)** Conserved motifs in TaADFs. The motifs, numbered 1–14, are displayed in different colored boxes. Motifs correspond to the ADF-H domain region are shown in red.

The TaADF amino acid sequences were aligned and a phylogenetic tree was constructed using the MEGA 7.0 program (Figure 4A). A structural analysis of the *TaADF* genes indicated that 19 *TaADF* genes have three exons, whereas the remaining genes have two exons (Figure 4B). Additionally, 19 *TaADF* genes consist of a 150-bp exon at the C-terminus and a second exon comprising 247–289 bp. Thus, the exon lengths and positions appear to be highly conserved in wheat *TaADF* genes. A total of 14 conversed motifs were identified (motifs 1–14) (Figures 4C,D). Motifs 1, 2, and 5 are three conserved regions that form the ADF-H domain in all TaADF proteins, whereas motif 3 is present in the N-terminus of 23 TaADF proteins (Figure 4C). Alignment of the predicted TaADFs revealed that the ADF-H domain position and actin binding sites were conserved in all of the ADFs (Supplementary Figure 1). Additionally, our analysis indicated that closely related homologous TaADF genes in the A, B, or D sub-genomes are usually similar regarding their structures and encoded motifs, suggesting wheat TaADF genes were conserved during evolution.

### Analysis of *Cis*-Elements of *TaADF* Genes in Wheat

The *cis*-acting elements in the 1,500-bp upstream promoter region of the identified *TaADF* genes were predicted using the PlantCARE online program. Fifty-seven *cis*-acting elements related to cell cycle regulation, plant development, hormone responses, stress responses, and transcription are presented in Supplementary Figure 2. Many *cis*-acting elements were associated with responses to various hormones, including abscisic acid (ABRE), methyl jasmonate (CGTCA-motif and TGACG-motif), auxin (TGA-element and AuxRR-core), and gibberellin (P-box and GARE-motif). Some of the identified *cis*-acting elements may regulate the development of specific tissues such as the endosperm (GCN4-motif), seed (RY-element), and meristem (CAT-box). The promoters of 16 and 13 *TaADF* genes included *cis*-acting elements responsive to drought (MBS) and low temperature (LTR), respectively. The presence of multiple *cis*-acting elements in the *TaADF* promoters may be indicative of the diversity in the biological functions of the encoded proteins.

### Expression Profiles of *TaADF* Genes in Various Wheat Tissues

**FIGURE 5:**
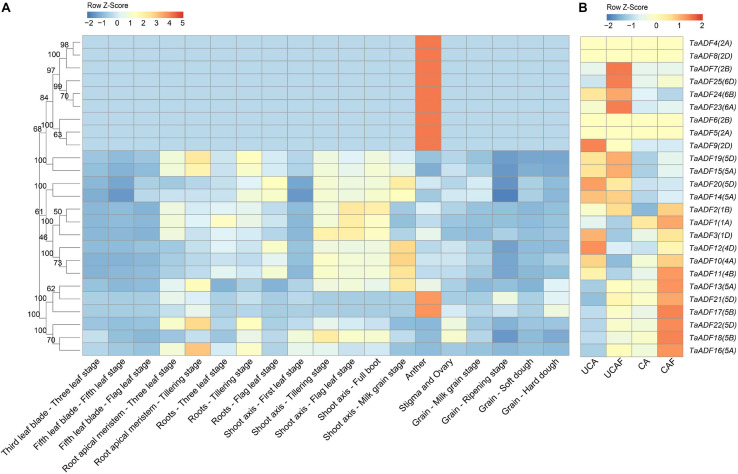
Expression patterns of 25 *TaADFs* in wheat. **(A)** Expression patterns of *TaADFs* in different tissues and growth stages. **(B)** Expression patterns of *TaADFs* under low temperature. Expression is scaled across genes (z score). CA: cold acclimation at 4°C for 28 days; UCA: un-cold acclimation at 20°C for 28 days; UCAF: UCA followed with −5°C for 1 day; CAF: CA followed with −5°C for 1 day.

To gain insights into the *TaADF* expression patterns in diverse wheat tissues, the available RNA-seq data for various wheat tissues across different developmental stages were obtained from the Wheat eFP database (Figure 5A). The *TaADF* expression levels varied among tissues at the same growth stage. At the flag leaf stage, the TPM of *TaADF15* was 113.68, 248.09, and 302.91 in the leaf blade, root, and shoot axis, respectively. Nine genes (*TaADF4/5/6/7/8/9/23/24/25*), located on chromosome 2 or 6, were highly expressed in the anther (TPM: 239.41–823.54), but were expressed at low or undetectable levels (TPM < 1) in the leaf blade, root apical meristem, root, shoot axis, and grain. Additionally, the expression of most *TaADF* genes in specific tissues varied substantially at different growth stages. For example, *TaADF15/16/19/18/22* expression levels in the root apical meristem and root were higher at the tillering stage than at the three-leaf stage. During the four examined grain developmental stages, the expression levels of these genes were highest and lowest at the milk grain stage and ripening stage, respectively. The tissue- and temporal-specific expression of *TaADFs* may help to clarify the complex functions of *TaADF* in various cellular processes.

The expression pattern of *TaADFs* were examined by RT-PCR in leaf and root at three-leaf stage (Supplementary Figure 3). The expression of homologous genes *TaADF15-5A* and *TaADF19-5D* were extremely high in both leaf and root, while the homologous genes *TaADF10-4A*, *TaADF11-4B*, and *TaADF12-4D*, showed significantly lower expression abundances. These results indicated that homologous *TaADF* genes in closely related clades appear to be expressed in different tissues similarity, implying they may be functionally similar.

### Expression Profiles of *TaADF* Genes Under Cold Conditions

To further evaluate the potential functions of ADFs in response to low temperatures, the ADF gene expression patterns under cold acclimation and freezing conditions were analyzed based on the fragment per kilobase of transcript per million reads mapped (FPKM). Results showed that *TaADFs* had differential expression under different temperature treatment. The expression of *TaADF13/16/17/18/21/22* was induced by the cold acclimation and freezing treatments, while the expression of *TaADF14/20* were decreased (Figure 5B). In total, seven DEGs were identified in three comparison (CA vs. UCA, CAF vs. CA and UCAF vs. UCA) (Supplementary Table 8). With the exception of *TaADF20*, the expression levels of all DEGs were up-regulated under the cold acclimation or freezing conditions. Significant changes to *TaADF13/20* expression were detected only in the CA vs. UCA. Up-regulated *TaADF17* expression was detected only in the CAF vs. CA. The *TaADF16/18* expression levels were up-regulated after the CA and UCA samples when exposed to freezing stress (CAF vs. CA and UCAF vs. UCA), with a greater change in expression in the samples that did not undergo the cold acclimation process. These results suggest that *TaADF16/17/18* contribute to the freezing tolerance of wheat plants acclimated to the cold.

To verify the *TaADF* expression patterns induced by the cold acclimation and freezing conditions, the expression levels of 10 *TaADF* genes were analyzed by RT-PCR. The consistency between the RT-PCR data and the RNA-seq data was reflected by the calculated correlation coefficient (*R*^2^ = 0.83) (Supplementary Figure 4). These results confirmed the accuracy of the RNA-seq results.

### Subcellular Localization of TaADFs

**FIGURE 6:**
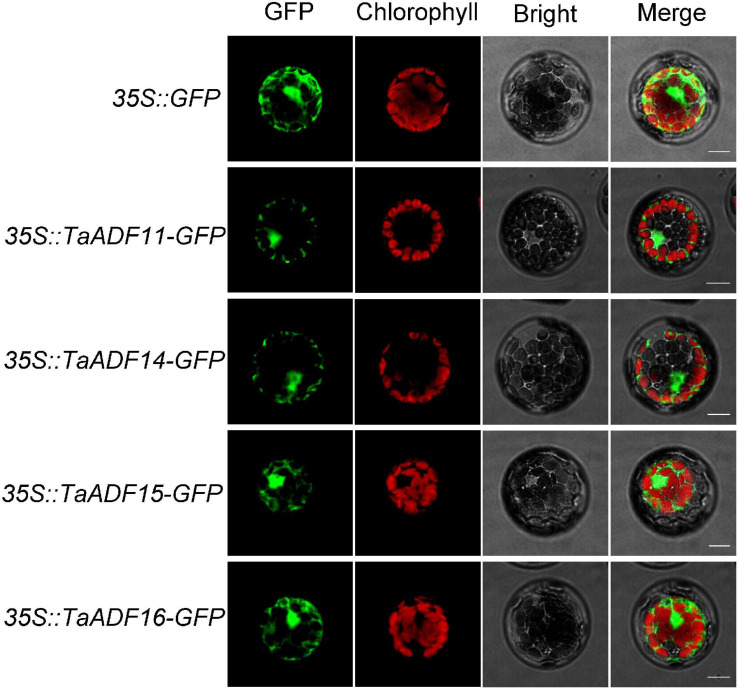
Subcellular location of TaADF11, TaADF14, *TaADF15*, and TaADF16 in *A. thaliana* protoplasts. Scale bars = 10 μm. 35S::GFP was used as a negative control.

Based on the predicted subcellular localizations, all the TaADFs are cytoplasmic proteins, whereas TaADF22 is present in the cytoplasm and nucleus (Table 1). To further confirm the prediction of subcellular localization of TaADFs, we constructed a fusion vector, which was transformed into *A. thaliana* protoplasts and observed by laser scanning confocal microscope (Figure 6). All the four proteins (TaADF11, TaADF14, TaADF15, and TaADF16) have strong fluorescence signal in transformed *A. thaliana* protoplasts, the four protein are localized in the cytoplasm and nucleus.

### Overexpression of *TaADF16* Enhanced the Freezing Tolerance of *Arabidopsis*

**FIGURE 7:**
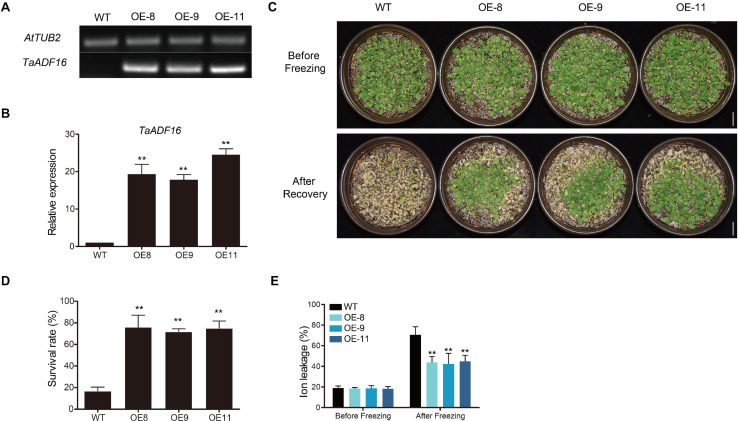
Overexpression of *TaADF16* enhances freezing tolerance in *Arabidopsis*. Detection of *TaADF16* mRNA in transgenic *Arabidopsis* using **(A)** Semi-quantitative RT-PCR and **(B)** RT-PCR; **(C)** Freezing tolerance of the transgenic *Arabidopsis*. Line bar: 2 cm. **(D)** Survival rate (%) and **(E)** Ion leakage (%) of WT and OE lines. Before freezing: three-week-old *Arabidopsis* seedlings under control; after freezing: three-week-old *Arabidopsis* seedlings under −5°C for 4 h and recovery at 22 °C for 4 days. Error bar indicated SD among at least three independent replicates. **P < 0.01 (Student’s *t*-test).

Among the *TaADF* genes, *TaADF16* was the most highly expressed (FPKM: CA 684.5, CAF 1837.33, UCAF 932.94) and up-regulated gene (CA vs. UCA 3.72-folds, CAF vs. CA, 1.56-folds, UCAF vs. UCA, 4.12-folds) under cold acclimation or freezing treatment (Figure 5B and Supplementary Table 8). Therefore, it was functionally characterized using transgenic *Arabidopsis* plants. Three transgenic lines with high *TaADF16* expression levels were selected for further analyses (Figures 7A,B). Before the freezing treatment, there were no obvious differences between the wild-type (WT) and *TaADF16*-overexpressing (OE) plants. Morphological changes consistent with freezing damage were detected in the WT and OE lines, but the damage was more severe in the WT plants (Figure 7C). The survival rate (%) of WT was 16.67%, whereas the survival rate of OE8, OE9, and OE11 lines were obviously higher (75.83%, 71.67, and 74.87%, respectively) after recovery (Figure 7D). The electrolyte leakage after the freezing treatment was significantly greater in the WT (70.65%) plants than in the OE lines (OE8 43.78%, OE9 42.28%, and OE11 44.87%) (Figure 7E), suggesting the cell membranes were more severely damaged in the WT plants than in the transgenic lines.

The activity of POD and SOD, as well as the soluble sugar content of the OE lines were similar to those in the WT before cold treatment, but the OE lines had higher POD and SOD activities and accumulated more soluble sugar than the WT plants after a 24-h incubation at 4°C (Supplementary Figures 5A–C). Following the cold treatment, the MDA content increased in both WT and OE lines, but MDA was significantly less abundant in the OE lines (OE8 3.38 μmol/g, OE9 2.80 μmol/g, and OE11 2.83 μmol/g) than in the WT plants (4.40 μmol/g) (Supplementary Figure 5D). Therefore, the overexpression of *TaADF16* appeared to enhance the freezing tolerance of *Arabidopsis* by modulating the scavenging of ROS and by altering the osmotic regulation.

To further investigate the regulatory effects of *TaADF16* in response to low-temperature stress, seven cold-responsive genes (*CBF1*, *CBF2*, *CBF3*, *COR15A*, *COR15B*, *COR47*, and *RD22*) were selected for a RT-PCR analysis. The basal and cold-induced expression levels of these genes were higher in the OE lines than in the WT plants (Supplementary Figure 6). These results suggest that TaADF16 overexpression induces the expression of cold-responsive genes, thus enhancing the cold resistance of transgenic plants.

## Discussion

The ADF gene family is relatively small in higher plants, with only 12, 12, 13, 11, and 14 members in rice, *Arabidopsis*, maize, tomato and poplar, respectively (Feng et al., 2006; Roy-Zokan et al., 2015; Khatun et al., 2016; Huang et al., 2020). In this study, we identified 25, 18, 12, 11, 8, and 5 ADF genes in the wheat, *T*. *dicoccoides*, *T. turgidum*, barley, *Ae*. *Tauschii*, and *T. urartu* genomes. The fact that more ADF genes were detected in wheat than in the other species may be attributed to the two rounds of polyploidization that occurred during wheat evolution (Marcussen et al., 2014). The presence of only a few *ADF* genes in the genomes of wheat relatives indicates that *TaADF* genes evolved after naturally occurring genomic hybridizations and fusions. The ADF gene family is believed to be structurally and functionally conserved in plants (McCurdy et al., 2001). Analyses of the phylogenetic relationships, gene structures, and encoded motifs indicated that closely related *TaADF* homologous in the A, B, or D sub-genomes have similar exon–intron structures and encode the same conserved motifs, which is consistent with the results of an earlier study (Feng et al., 2006). Our phylogenetic tree revealed that the ADF genes in the examined monocots are clustered in four main groups, with the ADF genes in the eudicot *Arabidopsis* distributed in subgroups I, II, and V. The phylogenetic relationships among the ADF genes from selected species were consistent with those described in published reports (Khatun et al., 2016; Huang et al., 2020). Accordingly, the ADF genes in the analyzed flowering plants likely evolved from a common ancestor.

Tandem and segmental duplications are considered to be the major driving force of gene family expansions during evolution (Cannon et al., 2004). Segmental duplications were revealed in this study as the main events responsible for the evolution of the TaADF gene family (Figure 1B). Similar events likely occurred in maize and tomato (Khatun et al., 2016; Huang et al., 2020). Therefore, we speculate that the ADF gene families in higher plants expanded primarily because of segmental duplications. The homologous ADF genes at the branch ends of each clade of sub-genome A, B, or D are likely the putative homoalleles of genes that evolved in *Ae*. *tauschii*, *T*. *dicoccoides*, and bread wheat (Figure 2B).

Tissue- and temporal-specific expression patterns of genes in growing plants usually reflect the differences in the biological functions of gene family members as well as the cross-talk among the associated pathways (Hu et al., 2018; Zhao et al., 2019a). Nine *TaADF* genes (*TaADF4/5/6/7/8/9/23/24/25*) were highly expressed in the anther, stigma, and ovary, but were expressed at low levels in the other examined tissues (Figure 5A). Similar results were reported for *PhADF1/2* in petunia (Mun et al., 2000), *SlADF1/2/10/11* in tomato (Khatun et al., 2016), and *ZmADF1/2/7/12/13* in maize (Huang et al., 2020). The actin genes in the Arabidopsis genome have been divided into the vegetative class (expressed predominantly in the leaves, roots, stems, petals, and sepals) and the reproductive class (highly expressed in pollen) (Meagher et al., 1999). Because ADF proteins interact with actin, researchers also classified the ADF genes into vegetative and pollen-specific groups (Mun et al., 2000). We predict that the *TaADF4/5/6/7/8/9/23/24/25* genes clustered in subgroup IV belong to the reproductive class, whereas the other *TaADF* genes are grouped in the vegetative class. The *TaADF14/15/19/20* genes were more highly expressed than the other *TaADF* genes in the vegetative class, implying these four *TaADF* genes are important for wheat growth.

In this study, we detected dynamic changes to *TaADF* expression levels during various plant growth and developmental stages. In tomato, *SlADF1/5/7/9* are highly expressed in immature fruit, whereas *SlADF3/11* expression levels peak in the mature fruit stage (Khatun et al., 2016). In the current study, in developing wheat grains, the expression of most *TaADF* genes peaked in the milk grain stage, markedly decreased in the ripening stage, and then increased in the soft dough and hard dough stages. These findings suggest the encoded TaADF proteins have similar regulatory effects on actin filaments in wheat plants. However, there were no obvious expression-level trends common to all *TaADF* genes during the development of other tissues (e.g., leaf, root apical meristem, root, and shoot axis). These observations have compelled us to investigate the complex ADF regulatory mechanisms underlying wheat growth.

Previous studies confirmed that the expression of ADF gene is induced in plants exposed to low temperatures (Kerr and Carter, 1990; Danyluk et al., 1996; Ouellet et al., 2001; Tang et al., 2016). The reorganization regulated by ADFs may influence various cytoskeletal-associated cell processes. In response to low-temperature stress, microtubules are more easily depolymerized in cold-acclimated rye root tip cells than in non-acclimated cells, and this depolymerization enhances the freezing tolerance of the root tips (Kerr and Carter, 1990). We identified six *TaADF* genes (*TaADF13/16/17/18/21/22*) with significantly up-regulated expression under cold acclimation or freezing conditions, which is consistent with the cold-induced changes in *SlADF2/11* expression in tomato (Khatun et al., 2016). However, *TaADF20* expression was down-regulated by cold stress. This phenomenon might be explained by the antagonistic relationships among ADFs. For example, in Arabidopsis, *AtADF9* adversely affects *AtADF1* by regulating its ability to depolymerize actin, whereas the opposite effect is observed when *AtADF9* and *AtADF1* are ectopically expressed in tobacco cells (Tholl et al., 2011). Notably, *TaADF16/18/22* expression levels were low during all examined wheat growth stages, but were highly up-regulated by low-temperature stress. Accordingly, to cope with cold conditions, *TaADF* gene expression is induced in wheat plants, thereby increasing the remodeling of actin. These findings reveal the complexity of the TaADF regulatory mechanism under cold conditions. Osmotic and ROS homoeostasis is essential for plant cold tolerance (Zuo et al., 2019). In our study, the overexpression of *TaADF16* increased the freezing tolerance of Arabidopsis plants, likely because of the positive effects on ROS scavenging and osmotic regulation (Supplementary Figure 5). Furthermore, the expression of cold-responsive genes was induced in the transgenic *Arabidopsis* (Supplementary Figure 6), suggesting that *TaADF16* may regulate cold tolerance by interacting with ICE-CBF-related genes. However, to more comprehensively characterize the relationship between the remodeling of the actin cytoskeleton and wheat responses to cold stress, additional ADF genes may need to be identified and functionally annotated.

## Conclusion

In this study, we identified 25 *ADF* genes in wheat. Based on the protein sequence alignment, 117 ADFs from wheat and the other analyzed species were clustered into four main groups. Segmental duplications during evolution were important for the expansion of the *TaADF* gene family. Analyses of the phylogenetic relationships, gene structures, and encoded motifs suggested that TaADF were conserved during evolution. The tissue- and temporal-specific expression patterns of *TaADF* genes were revealed in this study. Nine genes preferentially expressed in the anther (*TaADF4/5/6/7/8/9/23/24/25*) are likely associated with pollen development. Additionally, we identified seven differentially expressed *TaADF* genes after low-temperature treatments. Specifically, the expression of homologous genes *TaADF16/18/22* were considerably induced by cold stress, implying these genes are critical for the freezing tolerance of wheat. Overexpression of *TaADF16* substantially increased the tolerance of transgenic plants to freezing stress because of the associated effects on the cell membrane and ROS homeostasis, as well as the CBF/DREB pathway genes. These results provide new insights into the regulatory functions of TaADF proteins related to wheat responses to low temperature, and provides candidate gene resources for breeding new wheat varieties with enhanced freezing tolerance.

## Data Availability Statement

Publicly available datasets were analyzed in this study. This data can be found here: NCBI GEO database:GSE135474 (https://www.ncbi.nlm.nih.gov/geo/query/acc.cgi?acc=GSE135474).

## Author Contributions

YZ and XY conceived the project, set the scientific objectives, and revised the manuscript. KX, SiZ, HL, WW, and ShZ conducted the experiments and analyzed the data. KX wrote the manuscript. All authors discussed the results as well as read and approved the final manuscript for publication.

## Conflict of Interest

The authors declare that the research was conducted in the absence of any commercial or financial relationships that could be construed as a potential conflict of interest.

## Footnotes

1 http://plants.ensembl.org/index.html

2 http://pfam.xfam.org/

3 https://www.ncbi.nlm.nih.gov/cdd/

4 http://smart.embl-heidelberg.de/

5 https://web.expasy.org/protparam/

6 http://www.csbio.sjtu.edu.cn/bioinf/plant-multi/

7 https://www.uniprot.org/

8 http://gsds.cbi.pku.edu.cn/

9 http://meme-suite.org/

10 http://bioinformatics.psb.ugent.be/webtools/plantcare/html/

11 https://github.com/tanghaibao/jcvi/wiki/MCscan-(Python-version)

12 http://bar.utoronto.ca/efp_wheat/cgi-bin/efpWeb.cgi

## Supplementary Material

Supplementary Figure 1 Multiple alignments of TaADFs. The solid arrow and open arrow indicate the binding site of G-actin (ADFs shown in red) and F-actin (all the 25 ADFs), respectively. The red solid line indicates the ADF-H domain position.

Supplementary Figure 2 Analysis of *cis*-acting elements in TaADFs.

Supplementary Figure 3 Analysis of expression profiles of TaADF genes in leaf (**A**) and root (**B**) by RT-PCR. Error bar indicated SD among at least three independent replicates, and the values differed significantly when *P* < 0.05. Varied letters meant a significant difference.

Supplementary Figure 4 Confirmation of RNA-seq results by RT-PCR. (A) Expression patterns of *TaADFs* by RNA-seq and RT-PCR. (B) Correlation between RNA-seq and RT-PCR for selected TaADF genes. The relative expression of *TaADFs* in crown of three leaf stage seedlings (TL) was set as the control. Three biological replicates for each sample were performed and bars represent the SD. CA: cold acclimation at 4°C for 28 days; UCA: un-cold acclimation at 20°C for 28 days; UCAF: UCA followed with −5°C for 1d; CAF: CA followed with −5°C for 1d.

Supplementary Figure 5 Analysis of SOD activity (**A**), POD activity (**B**), MDA content (**C**), and soluble sugar content (**D**) in WT and OE lines of *Arabidopsis*. Before cold: three-week-old *Arabidopsis* seedlings under control (22°C); after cold: three-week-old *Arabidopsis* seedlings under 4°C for 24 h. Error bar indicates SD among at least three independent replicates. ^∗∗^*P* < 0.01 (Student’s *t*-test).

Supplementary Figure 6 Expression levels of seven cold response genes in WT and OE lines before or after cold stress. Before cold: three-week-old *Arabidopsis* seedlings under control; after cold: three-week-old *Arabidopsis* seedlings under 4°C for 24 h. Error bar indicates SD among at least three independent replicates. ^∗∗^*P* < 0.01 (Student’s *t*-test).

Supplementary Table 1 The data of genome sequences used in this study.

Supplementary Table 2 Specific primers used in this study.

Supplementary Table 3 The information of ADF protein of different plant species in this study.

Supplementary Table 4 Sequence similarities among the TaADF proteins.

Supplementary Table 5 Ka/Ks ratios of collinearity *TaADF* genes in wheat.

Supplementary Table 6 The number of ADF proteins of different subgroups in phylogenetic tree.

Supplementary Table 7 ADF collinear gene pairs between wheat and other selected species.

Supplementary Table 8 The log2 (fold change) of differently expressed genes (DEGs) under cold stresses.
