# Supplementary figures and images for: Genome-Wide Identification and Low Temperature Responsive Pattern of Actin Depolymerizing Factor (ADF) Gene Family in Wheat (Triticum aestivum L.)
Source: Front Plant Sci. 2021 Feb 24;12:618984. doi: 10.3389/fpls.2021.618984 (PMC7943747; doi:10.3389/fpls.2021.618984)

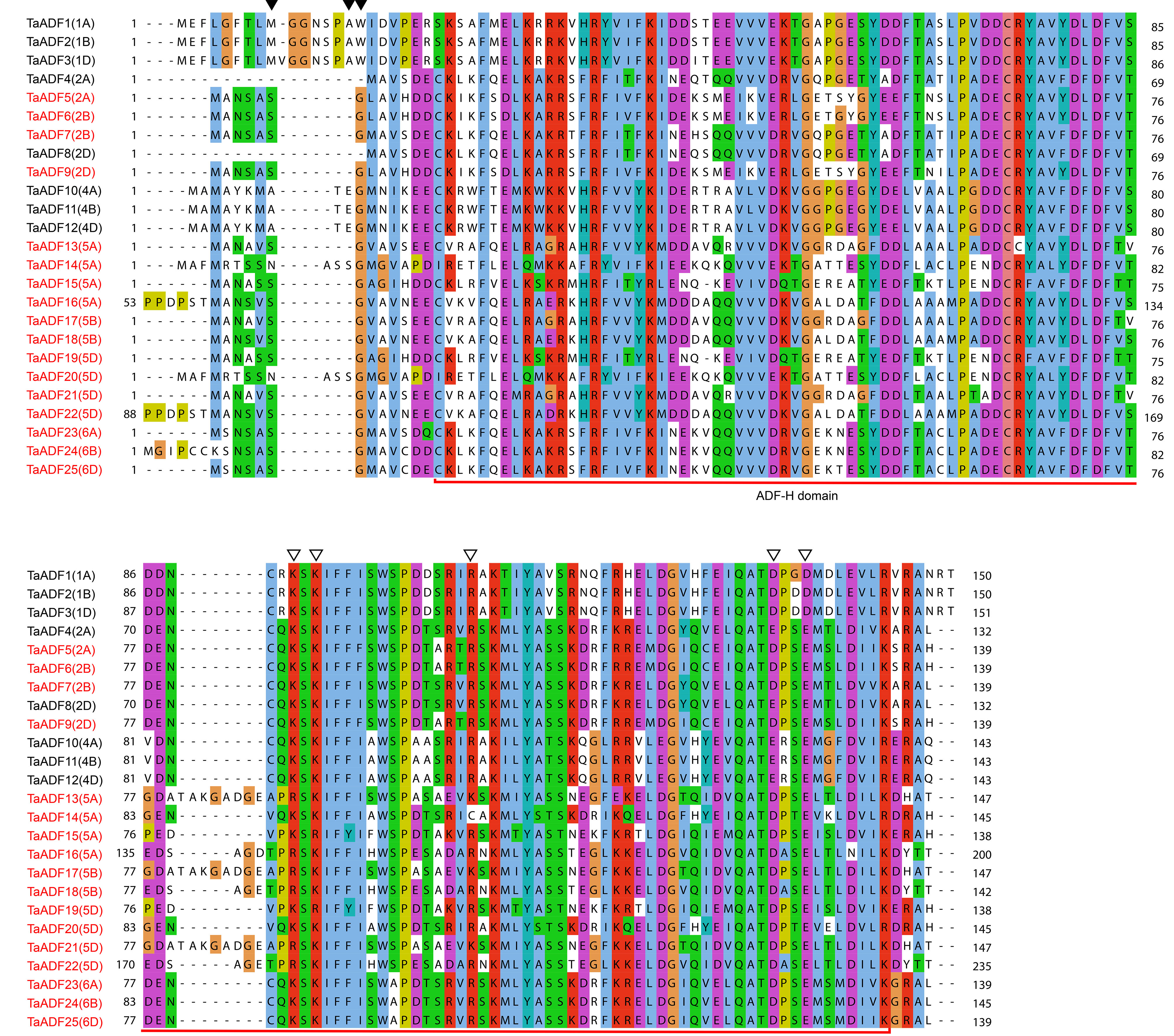

Supplement: Supplementary Figure 1 — Multiple alignments of TaADFs. The solid arrow and open arrow indicate the binding site of G-actin (ADFs shown in red) and F-actin (all the 25 ADFs), respectively. The red solid line indicates the ADF-H domain position. [file Image_1.JPEG]

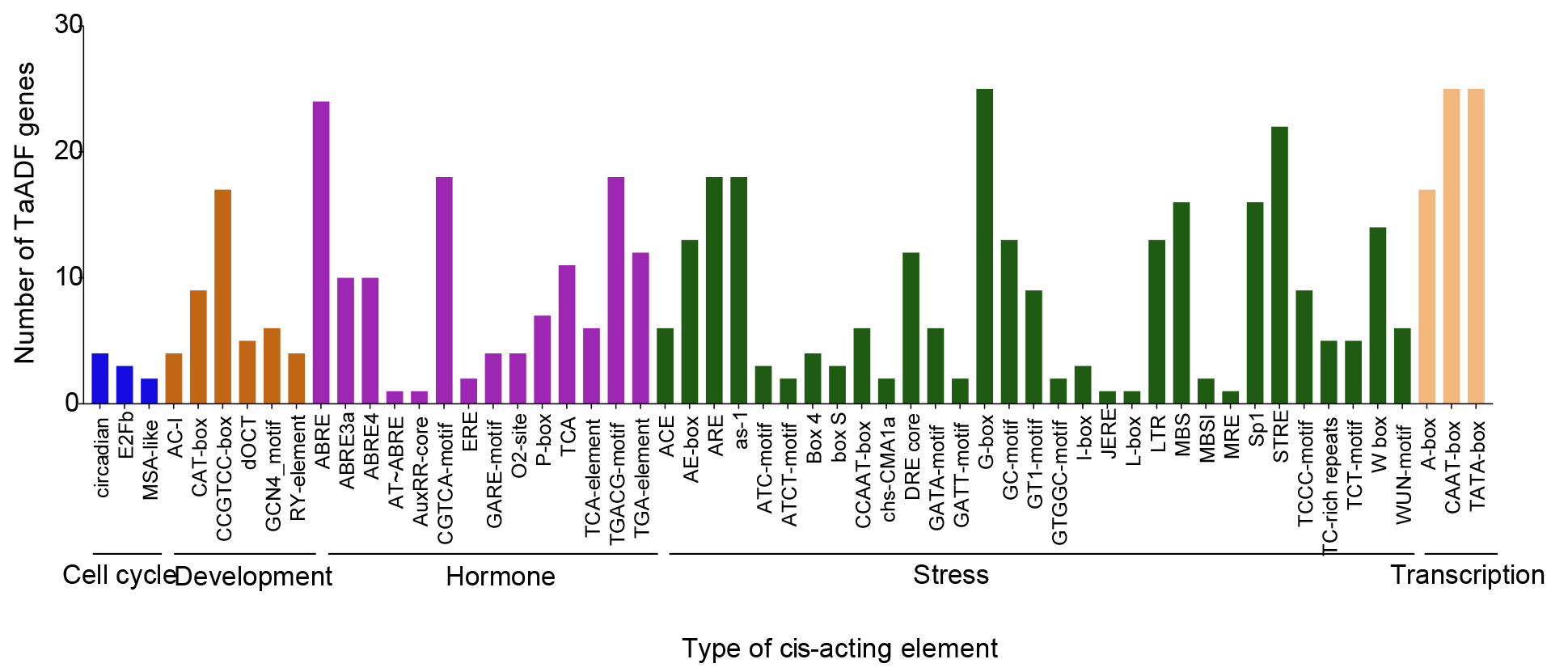

Supplement: Supplementary Figure 2 — Analysis of cis-acting elements in TaADFs. [file Image_2.JPEG]

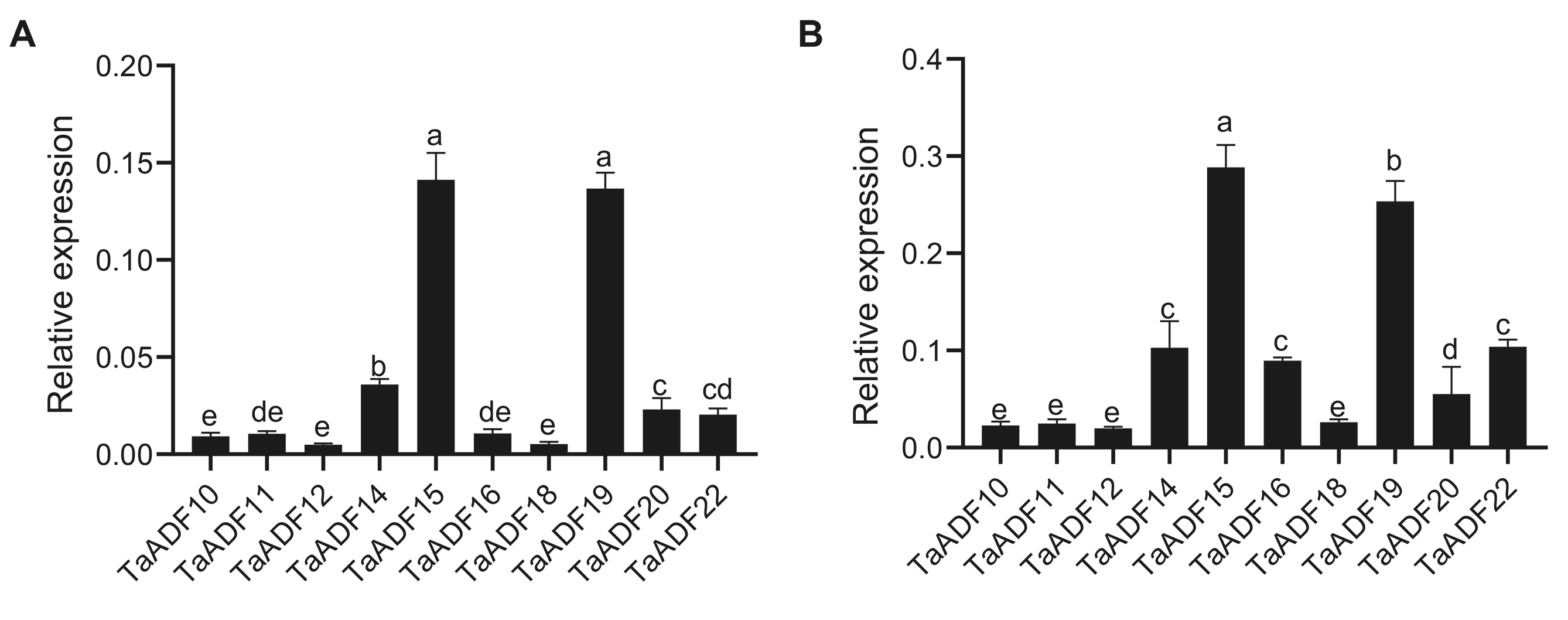

Supplement: Supplementary Figure 3 — Analysis of expression profiles of TaADF genes in leaf (A) and root (B) by RT-PCR. Error bar indicated SD among at least three independent replicates, and the values differed significantly when P < 0.05. Varied letters meant a significant difference. [file Image_3.JPEG]

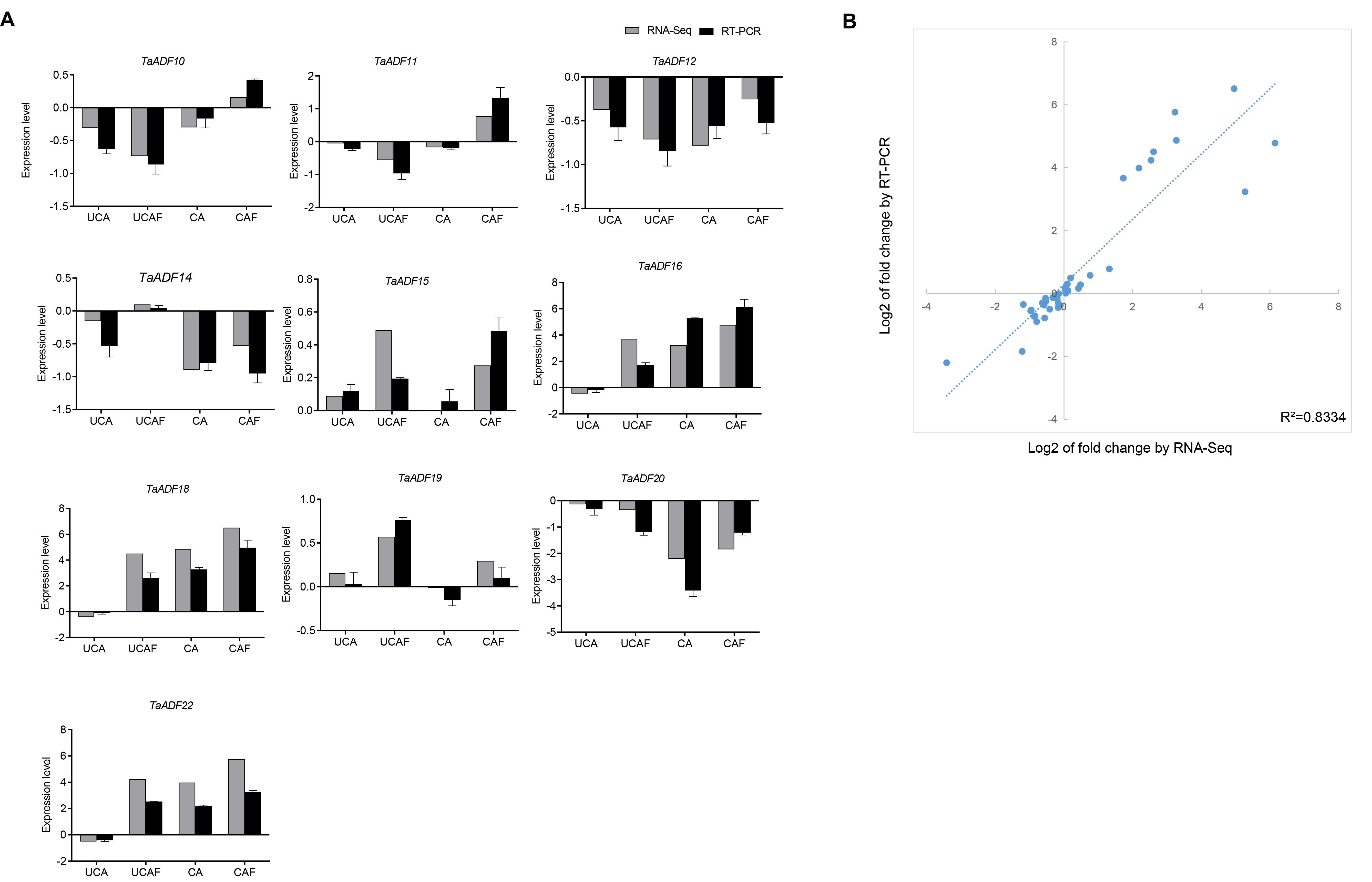

Supplement: Supplementary Figure 4 — Confirmation of RNA-seq results by RT-PCR. (A) Expression patterns of TaADFs by RNA-seq and RT-PCR. (B) Correlation between RNA-seq and RT-PCR for selected TaADF genes. The relative expression of TaADFs in crown of three leaf stage seedlings (TL) was set as the control. Three biological replicates for each sample were performed and bars represent the SD. CA: cold acclimation at 4°C for 28 days; UCA: un-cold acclimation at 20°C for 28 days; UCAF: UCA followed with −5°C for 1d; CAF: CA followed with −5°C for 1d. [file Image_4.JPEG]

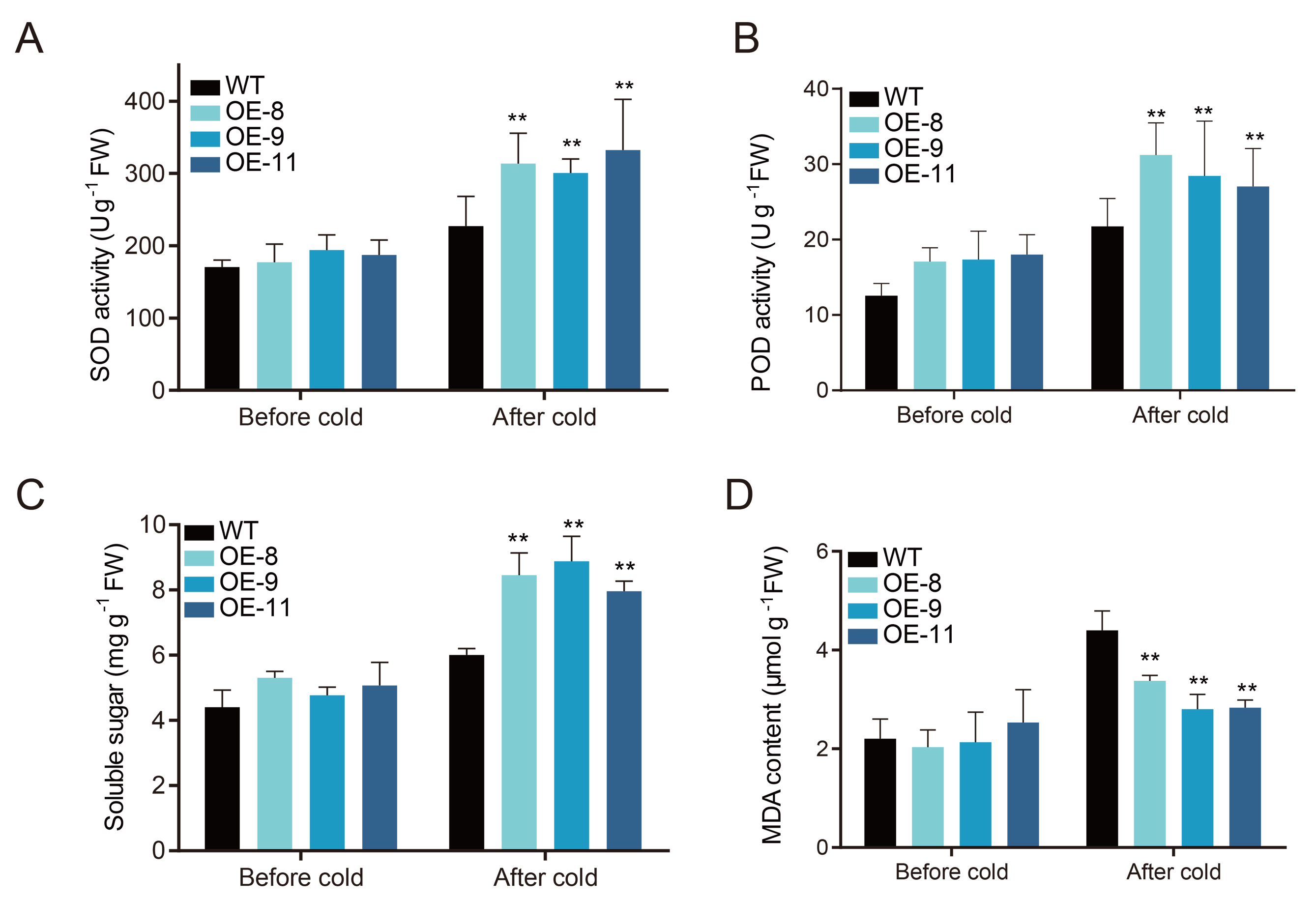

Supplement: Supplementary Figure 5 — Analysis of SOD activity (A), POD activity (B), MDA content (C), and soluble sugar content (D) in WT and OE lines of Arabidopsis. Before cold: three-week-old Arabidopsis seedlings under control (22°C); after cold: three-week-old Arabidopsis seedlings under 4°C for 24 h. Error bar indicates SD among at least three independent replicates. ∗∗P < 0.01 (Student’s t-test). [file Image_5.JPEG]

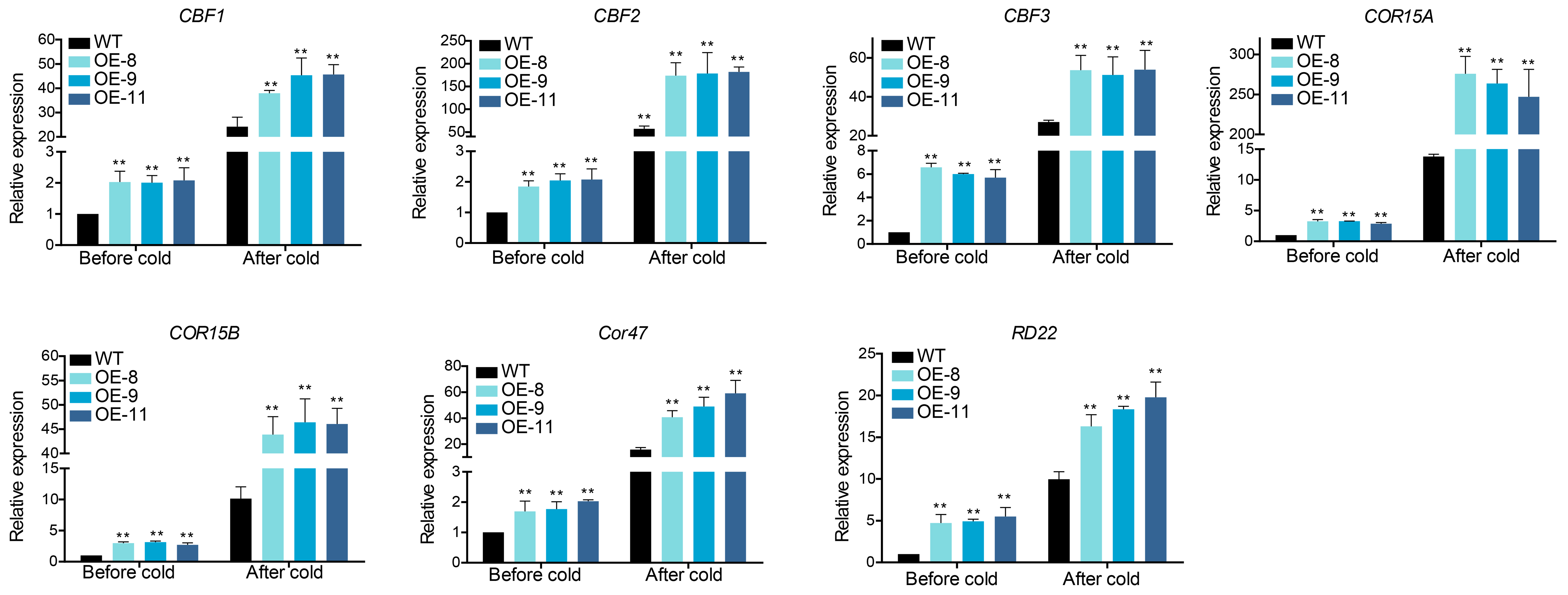

Supplement: Supplementary Figure 6 — Expression levels of seven cold response genes in WT and OE lines before or after cold stress. Before cold: three-week-old Arabidopsis seedlings under control; after cold: three-week-old Arabidopsis seedlings under 4°C for 24 h. Error bar indicates SD among at least three independent replicates. ∗∗P < 0.01 (Student’s t-test). [file Image_6.JPEG]
